# Anionic Dye Removal Using a Date Palm Seed-Derived Activated Carbon/Chitosan Polymer Microbead Biocomposite

**DOI:** 10.3390/polym14122503

**Published:** 2022-06-20

**Authors:** Hani Hussain Sait, Ahmed Hussain, Mohamed Bassyouni, Imtiaz Ali, Ramesh Kanthasamy, Bamidele Victor Ayodele, Yasser Elhenawy

**Affiliations:** 1 Department of Mechanical Engineering, Faculty of Engineering Rabigh, King Abdulaziz University, Rabigh 21911, Saudi Arabia; ahmad@neduet.edu.pk; 2 Department of Chemical and Materials Engineering, Faculty of Engineering Rabigh, King Abdulaziz University, Rabigh 21911, Saudi Arabia; inabi@kau.edu.sa (I.A.); rsampo@kau.edu.sa (R.K.); 3 Department of Chemical Engineering, Faculty of Engineering, Port Said University, Port Fouad 42526, Egypt; 4 Department of Chemical Engineering, Universiti Teknologi PETRONAS, Seri Iskandar 32610, Malaysia; ayodelebv@gmail.com; 5 Department of Mechanical and Power Engineering, Faculty of Engineering, Port Said University, Port Fouad 42526, Egypt; dr_yasser@eng.psu.edu.eg

**Keywords:** activated carbon, microwave radiation, chitosan, microbeads, adsorption, isothermal models, direct dye removal

## Abstract

The discharge of textile wastewater into aquatic streams is considered a major challenge due to its effect on the water ecosystem. Direct blue 78 (DB78) dye has a complex structure. Therefore, it is difficult to separate it from industrial wastewater. In this study, carbon obtained from the pyrolysis of mixed palm seeds under different temperatures (400 °C and 1000 °C) was activated by a thermochemical method by using microwave radiation and an HCl solution in order to improve its adsorption characteristics. The generated activated carbon was used to synthesize a novel activated carbon/chitosan microbead (ACMB) for dye removal from textile wastewater. The obtained activated carbon (AC) was characterized by a physicochemical analysis that included, namely, particle size, zeta potential, SEM, EDX, and FTIR analyses. A series of batch experiments were conducted in terms of the ACMB dose, contact time, pH, and activated carbon/chitosan ratios in synthetic microbeads for enhancing the adsorption capacity. A remarkable improvement in the surface roughness was observed using SEM analysis. The particle surface was transformed from a slick surface with a minor-pore structure to a rough surface with major-pore structure. The zeta potential analysis indicated a higher improvement in the carbon surface charge, from −35 mv (before activation) to +20 mv (after activation). The adsorption tests showed that the dye-removal efficiency increased with the increasing adsorbent concentration. The maximum removal efficiencies were 97.8% and 98.4% using 3 and 4 g/L of AC_400°C_ MB-0.3:1 and AC_1000°C_ MB-0.3:1, respectively, with initial dye concentrations of 40 mg/L under acidic conditions (pH = 4–5), and an optimal mixing time of 50 min. The equilibrium studies for AC_400°C_ MB-0.3:1 and AC_1000°C_ MB-0.3:1 showed that the equilibrium data best fitted to the Langmuir isothermal model with R^2^ = 0.99. These results reveal that activated carbon/chitosan microbeads are an effective adsorbent for the removal of direct blue 78 dye and provide a new platform for dye removal.

## 1. Introduction

Water is considered the most effective and central substance in human life. Due to its major applications in various industries, water is exposed to several pollutants and can therefore be contaminated. In recent years, water pollution has become a major threat to water ecology, and thus the extraction of pollutants from industrial wastewater before discharging into water streams is essential [1]. Industrial wastewater mostly contains organic and nonorganic contaminates that can be toxic to humans and aquatic life [2]. Amongst several pollutants, synthetic dyes are considered highly toxic and cancerogenic materials because they have complex aromatic structures that lead to difficult decomposition and separation [3]. Several industries, such as cosmetics, paper, textiles, printing, and pharmaceuticals, discharge large amounts of wastewater, which contain dyes and other toxic chemicals [4]. It was reported that 50 × 10^3^ tons of organic dyes are disposed worldwide every year [5]. Synthetic dyes are classified into three categories: anionic (direct, acidic, and reactive), cationic (all base dyes), and nonanionic (dispersed dyes) [6,7]. It is stated that direct dye has a highly harmful effect on the environment because it has a high water solubility, which makes it extremely difficult to extract using traditional methods [8]. One of its characteristics is the capacity to spread color to a given substrate due to its molecular structure, which contains chromophoric groups. These colors disrupt the water bodies aesthetically, contributing to a decline in the rate of photosynthesis and dissolved oxygen levels, and affecting the entire aquatic biota [9].

Several methods have been investigated for dye removal from industrial wastewater, such as nanofiltration, ozonation, flocculation, adsorption, reverse osmosis, chemical oxidation, and electrochemical, biological, and photocatalytic degradation [10,11,12,13,14,15]. However, these treatment technologies have shown a number of limitations and disadvantages, including a lower validation of the removal of different pollutants, high costs, high reagent requirements, and the generation of toxic waste products, which require further safe disposal [8]. From these techniques, adsorption is considered the most versatile method, and it has received a lot of attention due to its advantages, such as flexibility, low-sludge production, low cost, efficiency, and high speed [16,17]. Many adsorbents have been used efficiently for dye removal from industrial wastewater, such as activated carbon, chitosan, fly ash, and zeolites [18,19,20].

Activated carbon (AC) has been investigated and used as a potential adsorbent in several processes, such as the treatment of industrial effluents [21], groundwater treatment [22], and dye removal [23]. AC confirms the major adsorption in the gas and liquid phases due to its high micropore volume (V_mic_), large specific surface area (S_BET_), favorable pore size distribution, thermal stability, capability of high-speed adsorption, and low acid/base reactivity [24]. The high cost of the AC production process is the most significant challenge for commercial manufacturers, and the use of inexpensive raw materials with high carbon contents and low levels of inorganic compounds to produce low-cost AC has been a focus of research efforts in recent years. Agricultural byproducts and waste materials, such as rice husks [25], coconut husks [26], and oil palm fibers [27], are among the low-cost precursors for the production of AC. Moreover, chitosan has been investigated for various applications due to its biocompatibility, biodegradability, and derivability from abundant and inexpensive biomass [28]. Chitosan and chitosan-based composite materials have been used as sorption materials with high efficiency [29]. Chitosan hydrogel beads were produced by lowering the degree of crystallinity by generating a gel with the purpose of raising chitosan’s adsorption capability. Several strategies, including chemical crosslinking with cross-linking agents on their surface, have been developed to improve the commercial applicability of chitosan beads as an adsorbent material [30].

This study aims to develop a novel microbead composite adsorbent from activated carbon (AC) and chitosan hydrogel. AC was generated from the pyrolysis of mixed date palm seeds (DPS) under temperatures of 400 °C and 1000 °C, followed by the activation process using microwave radiation. These novel microbeads were used efficiently as an adsorbent material for the direct blue 78 (DB78)-dye-removal process.

## 2. Materials and Methods

### 2.1. Preparation of Activated Carbon (AC)

Two carbon samples, obtained from the pyrolysis of mixed date palm seeds (DPS) under temperatures of 400 °C and 1000 °C, were used in this study [31]. The carbon samples were placed into 400 mL distilled water. The solution was stirred 4 h with a mixing speed of 200 RPM at 30 °C. Samples were washed, filtered using a Whatman filter paper, and left to dry at 110 °C for 12 h using a thermal dryer. In order to activate the carbon, the samples were subjected to a microwave-chemical-activation process. These samples were activated by immersing 15 g of carbon into 250 mL of an HCl solution, with a concentration of 7% wt/wt, and then the solution was placed into a domestic microwave (frequency: 2.45 GHz; power: 600 W) for a contact time of 6 min. At the end of the activation process, the mixture was filtrated using a Whatman filter paper, washed using distilled water, and left to dry at 110 °C for 24 h. Physical and chemical analyses were conducted to characterize the activated carbon.

### 2.2. Chitosan and Dye

**Figure 1:**
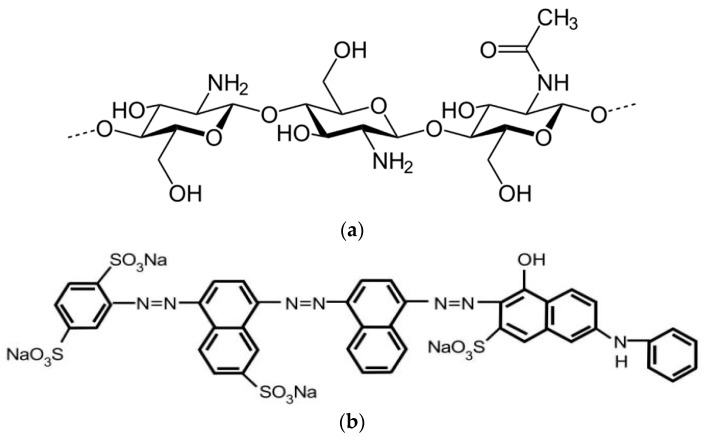
The chemical structure of (**a**) chitosan and (**b**) DB78 dye.

Chitosan is an amino-based polymer that is synthesized in vast amounts by the N-deacetylation of chitin. The characteristics of the chitosan used in this study are a white powder with a molecular weight range from 140 to 220 kDa, a degree of deacetylation (DAC) = 81.2%, viscosity = 36,000 cps, and a density of 0.15 g/mL. The chemical structure of chitosan is shown in Figure 1a. 

The direct blue 78 (DB78) dye had a relative molecular mass of 1059.95, a maximum wavelength of λ_max_ = 604 nm, and solubility up to 10 g/L at 25 °C. The direct blue 78 was selected for adsorption tests as it is widely used in the textile industry. The chemical structure of direct blue 78 is shown in Figure 1b. 

### 2.3. Preparation of Activated Carbon/Chitosan Microbeads (ACMB)

**Figure 2:**
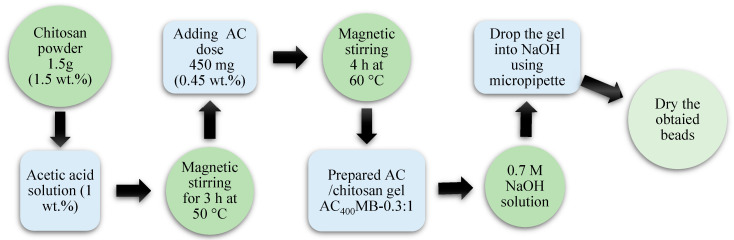
The preparation process for activated carbon/chitosan beads (AC_400°C_ MB-0.3:1).

Activated carbon/chitosan microbeads (ACMB) with different ratios of activated carbon to chitosan were synthesized. In AC_m_MB-z, m and z refer to the pyrolysis temperatures for carbon extraction (400 °C and 1000 °C) and the ratio of AC to chitosan, respectively. The adsorption studies were conducted using polymer-based composite materials with different mixing ratios: AC_m_MB-0.1:1, AC_m_MB-0.2:1, AC_m_MB-0.3:1, AC_m_MB-0.4:1, and AC_m_MB-0.5:1. In AC_400°C_ MB-0.3:1, the chitosan solution was prepared under magnetic stirring for 3 h by dissolving 1.5 g (1.5 wt.%) of chitosan powder in diluted acetic acid to form 100 mL of chitosan hydrogel. A total of 450 mg from activated carbon (pyrolysis temperature: 400 °C), representing 0.45 wt.%, was added to the formed hydrogel (45 mg of AC was added to 10 mL of chitosan hydrogel under magnetic stirring for 4 h and a temperature of 60 °C). The final prepared gel was dropped into a 0.7 M NaOH solution (contact time: 3 h) using a micropipette to form the beads. The formed beads were then washed using distilled water. Finally, the beads were oven-dried at 60 °C. The preparation process is illustrated in Figure 2.

### 2.4. Adsorption Studies

This study was conducted using the batch adsorption system (lab scale) on single-component synthetic wastewater. The activated carbon/chitosan microbead dose used in this study was 0.5–6.5 g. Synthetic wastewater with an initial concentration of 40 mg/L was mixed with ACMB at 200 RPM for the contact time (0–100) minimum at room temperature (25 ± 2 °C). The spectrophotometrically analysis was applied to determine the removal efficiency by measuring the dye concentration before and after the adsorption process at λ_max_ = 600 nm for DB78, as given in Equation (1):

(1) $$\%\left(R\right)=\frac{C_{O}-C}{C_{O}}\times 100$$

Equilibrium loading can be determined by using Equation (2):

(2) $$\mathrm{Equilibrium}\;\mathrm{loading},\;\mathrm{mg}/g\;=\left(C_{O}-C\right)\times V/m$$

where C_O_ and C are the initial and equilibrium concentrations of pollutant, respectively; V is the volume of solution (L); and m is the quantity of adsorbent (g).

### 2.5. Characterization of Materials

The activated carbon suspension was diluted in water and sonicated for 30 min in an ultrasonic bath at 4% (*w*/*v*). Model USC-1400 is one of a kind (40 kHz of ultrasound frequency). The Malvern 3000 Zetasizer NanoZS (Malvern Instruments, Malvern, UK) was used to measure the average particle size of produced activated carbon at 400 °C and 1000 °C. It measures the diffusion of particles moving under Brownian motion, and it translates the data to size and size distribution using dynamic light scattering. It is also used in laser doppler microelectrophoresis to provide an electric field to a dispersion of particles, which then move at a rate proportional to their zeta potential. The Smoluchowski algorithm was used to determine the particle size. Prior to adsorption studies, the samples were degasified at 200 °C for 4 h, and then the surface area was measured in the presence of N_2_ adsorption at −195.65 °C using surface-area analyzers (Autosorb-l-C-8, Quantachrome, Boynton Beach, FL, USA). By applying the BET (Brunauer–Emmett–Teller) equation to the adsorption data, the BET surface area for the sample was determined.

FTIR studies for all samples were conducted using a VERTEX 80v vacuum FTIR Spectrometer, Bruker corporation, Germany. The surface morphology and porous microstructure of samples were investigated by SEM analysis (TESCAN MIRA-High Resolution scanning electron microscope, Tescan Essence company, Brno, Czech Republic). 

Surface morphology and elemental analysis were performed using field-emission scanning electron microscopy (TESCAN MIRA-High Resolution scanning electron microscope, Tescan Essence company, Brno, Czech Republic) coupled with an energy-dispersive X-ray (EDX) (Oxford instrument nano analysis detector, UK).

The colorimetric analysis was carried out in this study using a LAMOTTE smart spectrophotometer v3 2000-01-MN, Washington Ave. Chestertown, MD, USA. 

## 3. Results and Discussion

### 3.1. Characterization of Adsorbents

The adsorbent samples were characterized by using a zeta sizer to measure the particle size. The zeta potential was used to measure the carbon particles’ net surface charge, and BET analysis was conducted to determine the surface area. The surface morphology was investigated by using SEM analysis. The elemental analysis was studied by using EDX analysis. The chemical function groups were determined by using FTIR analysis.

#### 3.1.1. Zeta Sizer Analysis

**Figure 3:**
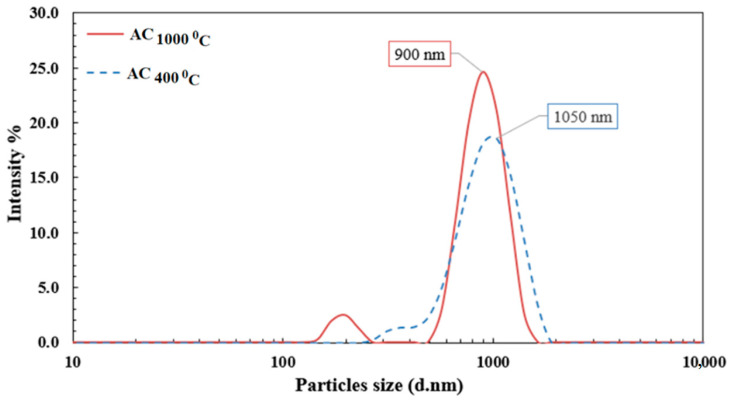
Particle-size-distribution analysis for activated carob samples.

Due to its major effect and great influence on the adsorption process, the particle size distribution of activated carbon was investigated. Figure 3 shows that 96.5% of the AC_400_ (activated carbon from a pyrolysis temperature of 400 °C) had average particle sizes less than 1.6 µm. For the AC_1000°C_ (activated carbon from a pyrolysis temperature of 1000 °C), 97.5% of the particle sizes were less than 1.2 µm. It was reported that there is an inverse relationship between the particle size and the adsorption capacity. The smaller the particle size, the higher the available surface area, which leads to a maximum adsorption capacity [32].

#### 3.1.2. Zeta Potential Analysis

**Figure 4:**
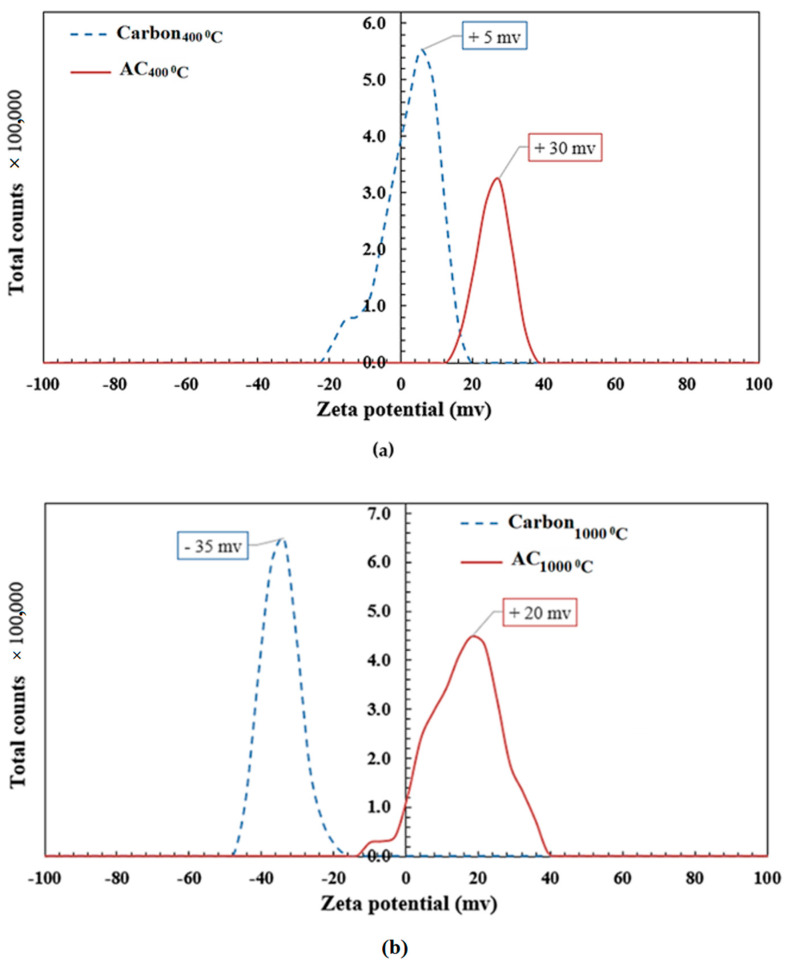
Effect of activation process on carbon surface charge of (**a**) carbon at 400 °C and (**b**) carbon at 1000 °C.

It was observed from the zeta potential analysis that the activation process using the HCl solution and microwave radiation has a remarkable effect on the surface charge of carbon particles. As illustrated in Figure 4a,b, carbon produced at high temperatures (1000 °C) has very hydrophobic behavior. However, due to the dehydration and deoxygenation of the palm seeds, it has reduced amounts of H- and O-containing functional groups. Surface groups can act as electron donors or acceptors, resulting in the creation of coexisting zones with a variety of characteristics that range from acidic to basic and from hydrophilic to hydrophobic. As a result, the ion-exchange capacity of carbon particles may be reduced. Biochar generated at lower temperatures (400 °C) has a more varied organic character due to the presence of aliphatic and cellulose-type structures. As a result, biochar’s structure looks to have less surface-functional-group content as the temperature increases. The surface charges of carbon particles were +5 mv and −35 mv, respectively, for carbon at 400 °C and 1000 °C. Figure 4a,b shows that the activation of carbon particles using acidic solution led to an increase in the positive charge to +30 mv and +20 mv for AC_400°C_ and AC_1000°C_, respectively. This improvement in the surface charge is attributed to the accumulation of hydrogen ions (H^+^) on the activated carbon particle surface after the thermochemical modification process using microwave radiation and the HCl solution (chemical agent).

#### 3.1.3. Energy-Dispersive X-ray (EDX) Analysis

**Figure 5:**
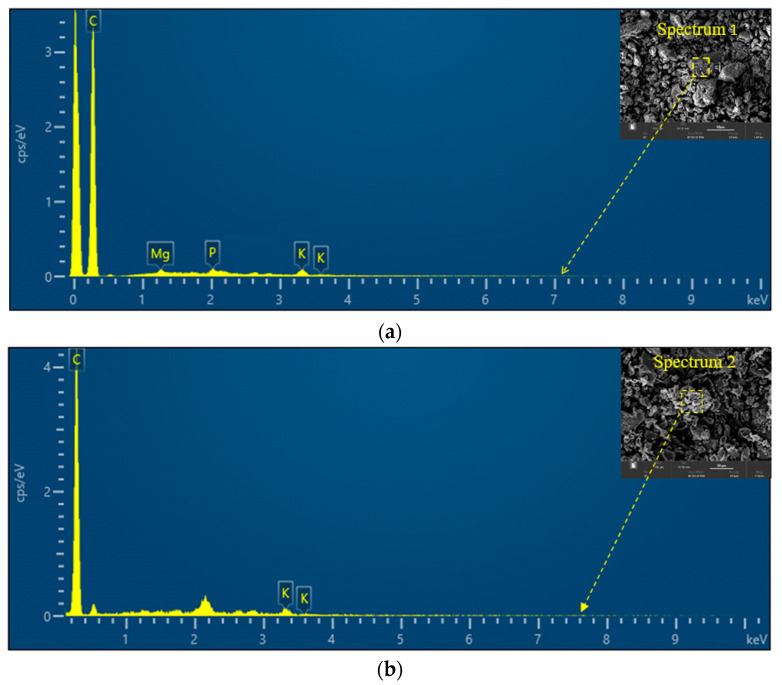
EDX analysis for charge: (**a**) activated carbon at 400 °C and (**b**) activated carbon at 1000 °C.

In order to determine the carbon content and presence of different elements in the two types of activated carbon samples (AC_400°C_ and AC_1000°C_), the energy-dispersive X-ray (EDX) analysis was conducted. Figure 5a shows peaks at ~0.2, 1.2, 2, and 3.5 KeV. These peaks are attributed to carbon (C), magnesium (Mg), phosphorus (P), and potassium (K), respectively. The elemental analysis for the AC_400°C_ sample indicated the presence of high carbon content (86.7%). For the AC_1000°C_, peaks are observed at 0.2 and 3.5 KeV. These peaks are attributed to carbon (C) and potassium (K), respectively. Furthermore, the elemental analysis for the AC_1000°C_ sample indicated the presence of a higher carbon content (97.4%) than the carbon contents for the AC_400°C_. These results are in good agreement with the reported data, as changes in the structure and physicochemical characteristics of biochar are significantly associated with the pyrolysis temperature. The temperature of pyrolysis has a significant impact on the physicochemical characteristics of biochar (e.g., surface area, pH, and functional groups). The surface area, carbonized fractions, pH, and volatile matter increased as the pyrolysis temperature increased, whereas the cation-exchange capacity and content of the surface functional groups decreased.

#### 3.1.4. Scanning Electron Microscopy (SEM) Analysis

**Figure 6:**
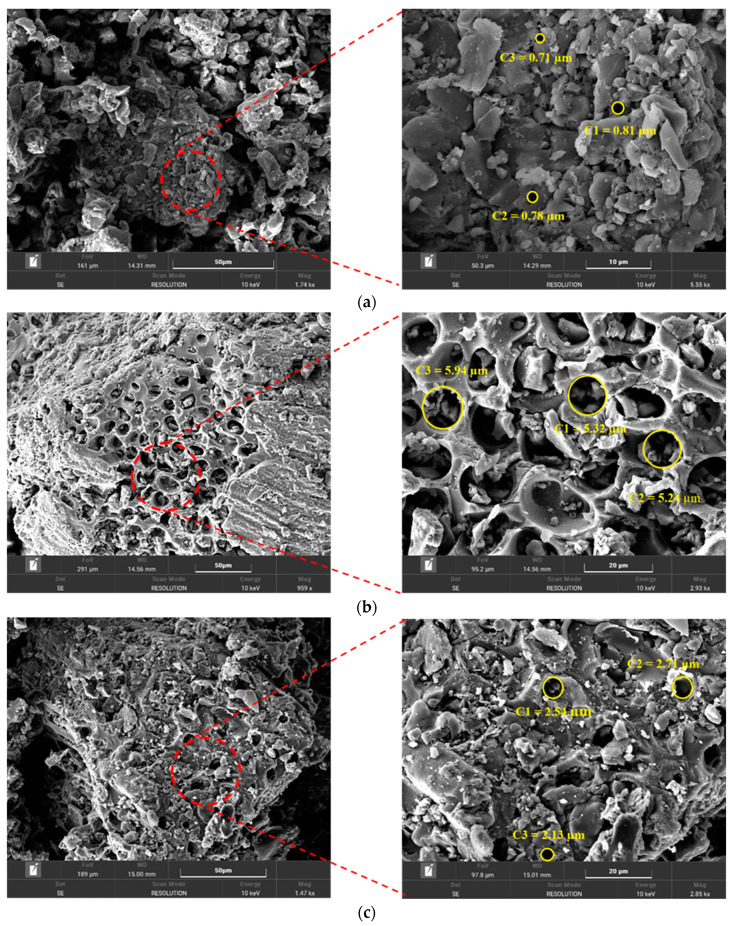

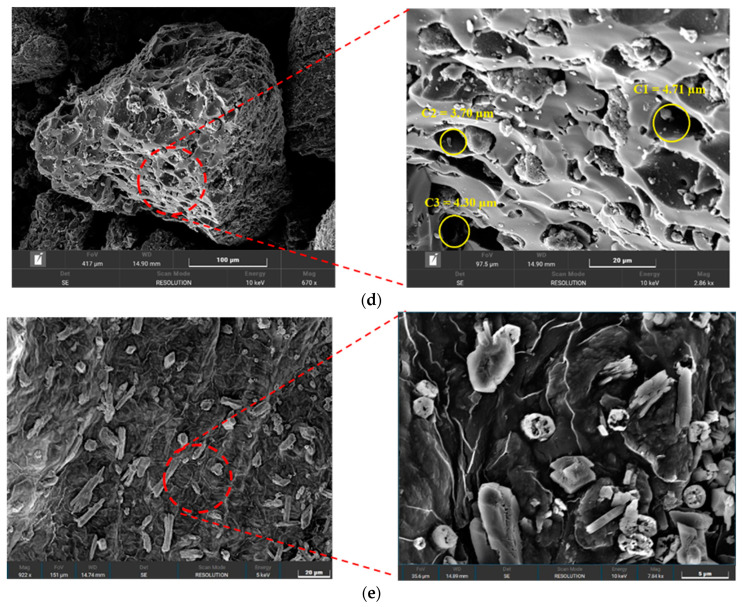
SEM analysis for (**a**) carbon at 400 °C, (**b**) activated carbon at 400 °C, (**c**) carbon at 1000 °C, (**d**) activated carbon at 1000 °C, and (**e**) AC_1000__°C_ MB-0.3:1.

An SEM analysis was conducted to study the impact of the thermochemical activation process using the HCl solution and microwave radiation on the carbon surface morphology and the pore’s structure. Figure 6a,c shows that the carbon obtained from the pyrolysis of date palm seeds (DPS) under temperatures of 400 °C and 1000 °C had a smooth and slick surface with a restricted pore structure. The two carbon samples had macropore diameters that ranged from 0.71–0.82 µm to 2.13:2.7 µm for carbon at 400 °C and 1000 °C, respectively. After the activation process, the carbon particles had a noticeable variation, as is shown in Figure 6b,d. The surface converted from a slick surface to an eroded rough surface, with a remarkable improvement in the porous structure. As a result of this major change in the surface morphology, the two activated carbon samples showed larger pore diameters. The pore size increased from 5.2 to 5.94 µm and from 3.70 to 4.71 µm at 400 °C and 1000 °C, respectively. This large improvement in the carbon physical characteristics can be attributed to the direct interaction between the microwave radiation and the particles inside the pressed compact material in the presence of HCl as a chemical agent. Figure 6e shows the distribution of the carbon particles (AC_1000°C_) in the chitosan matrix. The activated carbon was found to be capable of dispersing effectively in chitosan, and forming composite beads with no agglomerations. 

#### 3.1.5. FTIR Analysis

**Figure 7:**
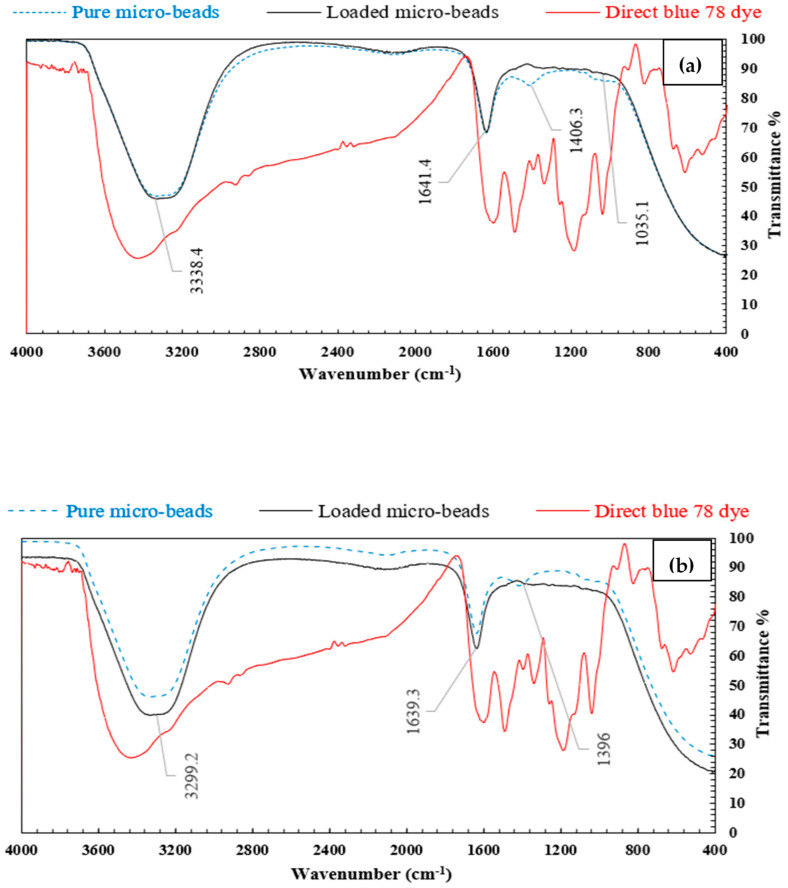
FTIR analysis for neat and loaded (**a**) AC_400__°C_ MB-0.3:1 and (**b**) AC_1000__°C_ MB-0.3:1.

In order to classify the main infrared (IR) bands of organics and determine the adsorption mechanism (physisorption or chemosorption), pure and loaded activated carbon/chitosan microbeads (AC_400__°C_ MB-0.3:1 and AC_1000__°C_ MB-0.3:1) were investigated by FTIR analysis. Figure 7a,b shows that the bands at 3338 cm^−1^ and 3299 refer to the bending stretch of the NH_2_ group. The peaks observed at 1641 cm^−1^ and 1639.3 can be attributed to the axial deformation of C=O of the acetamide group, which was assigned to the acetylated part of chitosan. The bands at 1406 cm^−1^ and 1035 cm^−1^ are assigned to the angular deformation of C–H of the CH_3_ group and to C–O binding and stretching, respectively. These results are close to those recorded for activated carbon/chitosan composites investigated elsewhere [33]. By comparing the two spectra (before and after adsorption), it was noticed that there was no change in the peaks. This means that no chemical bonds were formed within the adsorption process. The change in the peak intensity can be attributed to the electrostatic interaction between the anionic dye molecules and H^+^ ions that accumulated on the activated carbon surface and -NH_2_ groups on the surface of the chitosan.

#### 3.1.6. Surface Area

**Figure 8:**
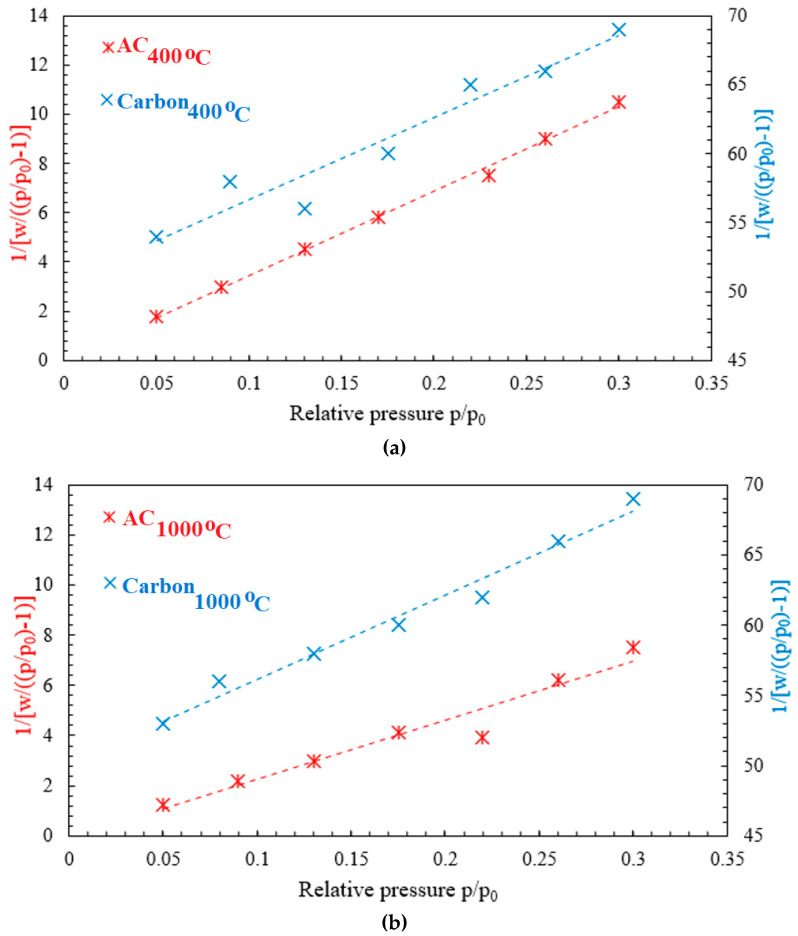
BET analysis for (**a**) carbon at 400 °C and activated carbon at 400 °C, and (**b**) carbon at 1000 °C and activated carbon at 1000 °C.

As shown in Figure 8, the BET surface area analysis was conducted in order to investigate the effect of the thermochemical activation process on the adsorption characteristics of carbon particles, such as the surface area and pore volume. The analysis showed that there was a higher improvement in the surface area and pore volume for carbon particles. For carbon at 400 °C, the BET surface area and pore volume increased from 32.5 m^2^/g and 0.023 cm^3^/g (before activation) to 99.9 m^2^/g and 0.037 cm^3^/g (after activation), respectively. Furthermore, for carbon at 1000 °C, the BET surface area and pore volume increased from 33.1 m^2^/g and 0.025 cm^3^/g (before activation) to 138 m^2^/g and 0.045 cm^3^/g (after activation), respectively. The results shown in Table 1 indicate that the applied activation process using the HCl solution and microwave radiation gas had a major effect on the BET surface area and pore volume for carbon particles.

### 3.2. Adsorption Tests

#### 3.2.1. Effect of Microbead Dose on DB78-Dye-Removal Efficiency

The doses of activated carbon/chitosan microbeads were varied in order to investigate their effects on the efficiency of the direct blue 78 dye removal. It was found that by increasing the microbead dosage (increasing the active adsorption sites), the equilibrium loading decreases and the removal efficiency increases until reaching the maximum efficiency, and then approximately reaches a fixed value. The experiments were conducted by varying the doses of AC_400°C_ MB-0.3:1 and AC_1000°C_ MB-0.3:1 from 0.5 to 6.5 g/L. The adsorption process was conducted at initial concentrations of DB78-dye synthetic solutions of 40 mg/L at an ambient temperature of 20 °C, a pH of 4–5, a stirring speed of 200 RPM, and a contact time of 40 min. 

**Figure 9:**
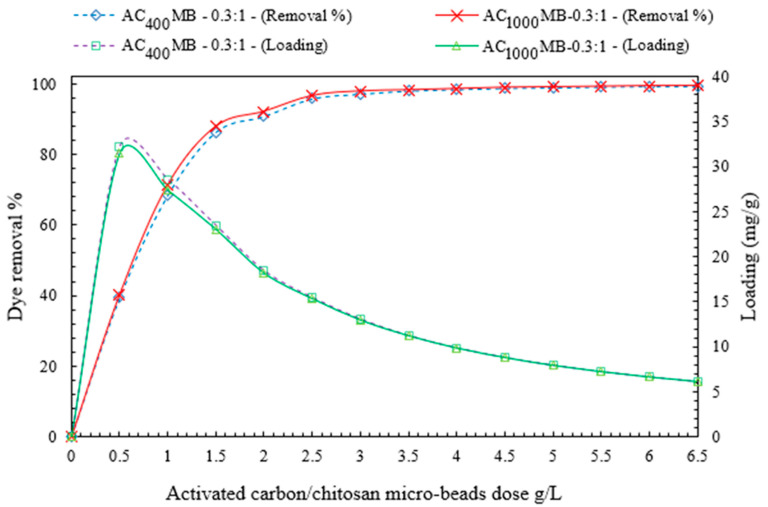
The effects of AC_400°C_ MB-0.3:1 and AC_1000 °C_ MB-0.3:1 doses on both dye-removal efficiency and equilibrium loading.

Figure 9 demonstrates the influence of the activated carbon/chitosan microbeads on both the dye-removal efficiency (%) and the equilibrium loading (mg/g). For AC_400 °C_ MB-0.3:1, the removal efficiency (97.8%) and equilibrium loading (13.1 mg/g) were obtained by using a AC_400°C_ MB-0.3:1 dose of 3 g/L for the solution with an initial concentration of 40 mg/L. The maximum equilibrium loading was reached at 31.5 mg/g. It was obtained by using a AC_400°C_ MB-0.3:1 dose of 0.5 g/L. 

Furthermore, for the AC_1000°C_ MB-0.3:1 experimental analysis, the removal efficiency (98.4%) and equilibrium loading (9.85 mg/g) were obtained by using a AC_1000°C_ MB-0.3:1 dose of 4 g/L for the solution with an initial concentration of 40 mg/L. The maximum equilibrium loading reached was 32.2 mg/g. It was obtained by using a AC_1000°C_ MB-0.3:1 dose of 0.5 g/L. It is worth noting that higher q values and lower C_eq_ values indicate that there is less dye in the solution and better adsorption.

#### 3.2.2. Effect of pH on DB78-Dye-Removal Efficiency

**Figure 10:**
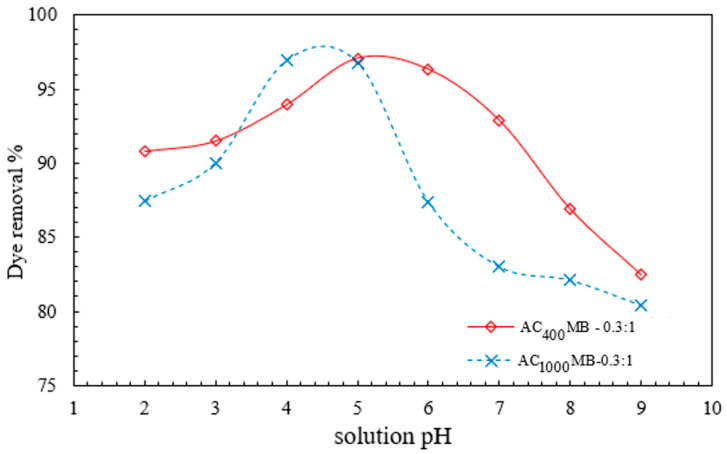
The effect of pH on DB78-dye-removal efficiency using AC_400°C_ MB-0.3:1 and AC_1000°C_ MB-0.3:1.

The effect of the initial pH of the dye solution was experimentally investigated under a pH range of from 2 to 9. The results are shown in Figure 10. For AC_400°C_ MB-0.3:1, the solution pH had a major effect on the chitosan adsorption behavior. AC_400°C_ MB-0.3:1 reaches its maximum removal efficiency (97%) under acidic conditions (pH = 5), in comparison with a 92% removal efficiency under alkaline conditions (pH = 9). Moreover, AC_1000°C_ MB-0.3:1 reaches its maximum removal efficiency (96.8%) under acidic conditions (pH = 4), in comparison with an 80% removal efficiency under alkaline conditions (pH = 9). The experiments were conducted under the following conditions: a temperature of 20 °C, a mixing speed of 200 RPM, a microbead dose of 3 g/L, and a contact time of 40 min. These results could be attributed to the major presence of hydrogen ions (H^+^) in the solution under acidic conditions. These ions accumulated on the activated carbon particles and improved their surface positive charge. This improvement in the surface charge increases the ability of activated carbon particles to attract the anionic molecules of direct blue 78 dye.

In addition, the swelling of chitosan powder into chitosan beads in the presence of acidic conditions will protonate the amine groups (NH_2_) into NH_3_^+^. This process will improve the electrostatic interaction between chitosan particles and dye ions, and enhance the activated carbon/chitosan microbead ability for anionic-dye removal.

#### 3.2.3. Effect of Contact Time on DB78-Dye-Removal Efficiency 

**Figure 11:**
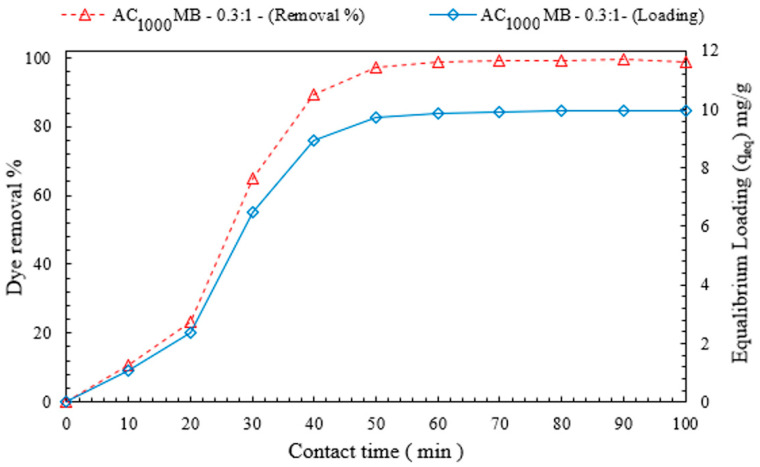
The effect of contact time on both DB78-dye-removal efficiency and equilibrium loading.

Figure 11 shows the influence of the contact time on the dye-removal efficiency and equilibrium loading. It was experimentally observed that the percentage of dye removal increases with the increase in the contact time. At a specific time, the microbead reached its maximum loading capacity (equilibrium loading q_e_). At contact time (50 min) and the AC_1000°C_ MB-0.3:1 dose (3 g/L), the equilibrium concentration decreased to 1.1 mg/L, with a dye-removal efficiency of 97%, and an optimum loading capacity of 9.7 mg/g for an initial dye concentration of 40 mg/L. These experiments were conducted under the following conditions: a temperature of 20 °C and a rate of mixing of 200 RPM.

#### 3.2.4. Effect of Activated Carbon/Chitosan Ratio on DB78 Dye Removal 

**Figure 12:**
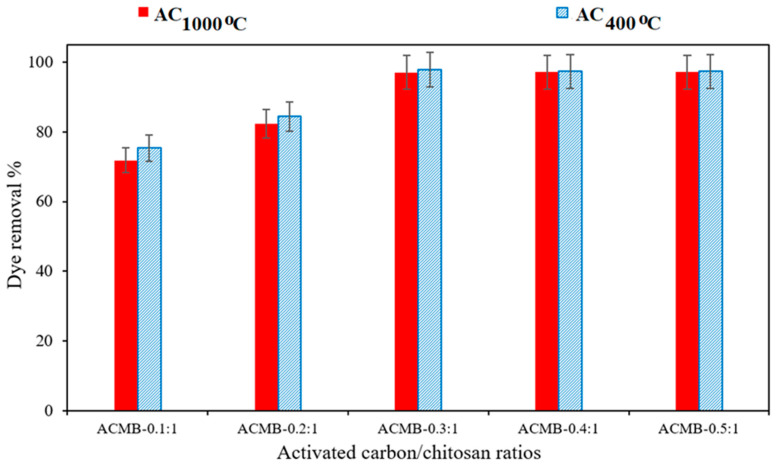
The effect of activated carbon/chitosan ratios on DB78-dye-removal efficiency.

**Figure 13:**
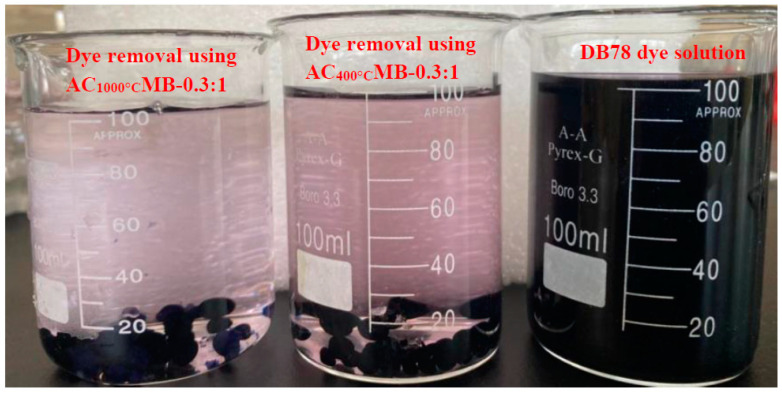
DB78 dye solution and dye removal using AC_1000°C_ MB-0.3:1 and AC_400°C_ MB-0.3:1.

To study the composition of the microbead production on the DB78-dye-removal efficiency, different ratios of AC and chitosan were applied (e.g., ACMB -0.1:1, ACMB-0.2:1, ACMB-0.3:1, ACMB-0.4:1, and ACMB-0.5:1). The initial dye concentration was 40 mg/L, the pH range was 4:5, the contact time was 40 min, the microbead dose was 3 g/L, and the stirrer speed was 200 RPM. The experimental results show that the dye-removal efficiency increases with the increase in the activated carbon loading up to 0.3:1. No significant improvement in the dye removal was found at higher activated carbon loads, as is shown in Figure 12. This can be attributed to a blockage of the internal porosities of chitosan by the incorporation of higher activated carbon loadings. Figure 13 shows the DB78 dye solution and dye removal using AC_400°C_ MB-0.3:1 and AC_1000°C_ MB-0.3:1. It was observed that there was a slight improvement in the efficiency of the dye removal using AC_1000°C_ compared with AC_400°C_. This result is in a good agreement with the EDX analysis, as a higher carbon content (97.4%) was found in the activated carbon produced at 1000 °C.

#### 3.2.5. Effect of Contact Time 

**Figure 14:**
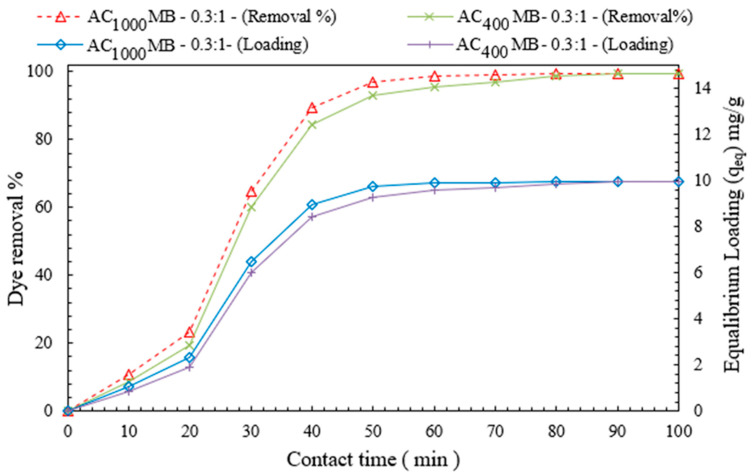
Effect of contact time on dye removal.

The influence of the contact time on the dye removal percentage and adsorbent loading was studied. The percentage of dye removed increased with the increase in the mixing time until the maximum removal efficiency was reached, as is illustrated in Figure 14. The adsorbent achieved its maximum loading capacity at this point (equilibrium loading q_e_). For a dye concentration of 40 mg/L of solution, a contact time of 50 min, and a 3 g/L adsorbent dose of AC_1000°C_ MB-0.3:1, the dye-removal efficiency was 99%, and the optimal loading capacity was 10 mg/g. When comparing AC_1000°C_ MB-0.3:1 with AC_400°C_ MB-3:1, a slight improvement in the dye removal was observed. This could be due to the AC_1000°C_ MB-3’s increased carbon content.

### 3.3. Adsorption Isotherm

**Figure 15:**
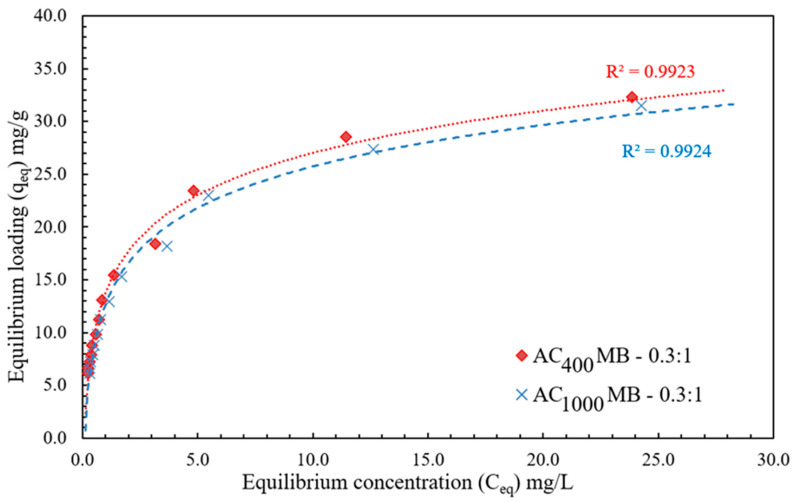
The adsorption isotherm for DB78 dye removal using AC_400°C_ MB-0.3:1 and AC_1000°C_ MB-0.3:1.

Figure 15 shows the adsorption isothermal curve. It indicates the amount of adsorbate (DB78 dye) by the activated carbon/chitosan microbead (AC_400°C_ MB-0.3:1 and AC_1000°C_ MB-0.3:1) q_eq_ against the adsorbate concentration in the liquid state (C_eq_). The initial concentration was 40 mg/L. These results are essential considerations in the design of adsorption systems. Moreover, the form of the equilibrium curve helps to describe other phenomena that are linked with the adsorption mechanism. The equilibrium curves are identified under four main classes according to the primary slope and subgroups that are described for each class based on the upper-part shapes and slope changes: (a) S curves, or vertical-orientation isotherms; (b) L curves, or normal or “Langmuir” isotherms; (c) H curves, or high-affinity isotherms; and (d) C curves, or constant-partition isotherms [34].

The initial shape of the equilibrium curve (L shape) in Figure 15 follows the basic premise that the higher the solute concentration, the greater the adsorption capacity, until the number of the adsorption site clearance is limited, and competition occurs between the solute molecules for the available sites. This isotherm type indicates that the adsorption occurs due to relatively weak forces, such as “van der Waals forces”. Several isothermal models (equations) are available, and the two important isotherms are selected in this study, which are, namely, the Freundlich and Langmuir isotherms.

**Figure 16:**
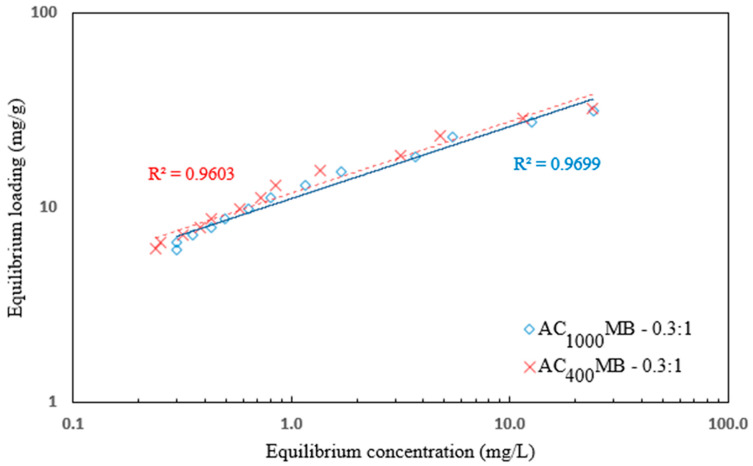
Freundlich isotherm for DB78 dye removal using AC_400°C_ MB-0.3:1 and AC_1000°C_ MB-0.3:1.

The Freundlich isotherm refers to the adsorption on a heterogeneous surface, and the adsorbed mass increased exponentially with an increase in the concentration [35]. This isotherm explains the equilibrium on heterogeneous surfaces and, hence, the capacity is not a presumed monolayer. In the liquid phase, this isotherm is given by Equation (3):

Q_e_ = K_f_ C_e_^nf^ (3)

where k_f_ is the Freundlich fixed value (k_f_ unit = mg/g, where n_f_ is the Freundlich exponent). This isotherm focused on integrating the role of the adsorbent (adsorbate–surface interactions). Figure 16 shows the application of equilibrium data according to the Freundlich isotherm. For AC_400°C_ MB-0.3:1, the Freundlich constant (k_f_) value was 11.86, and the heterogeneity factor (1/n_f_) value was 0.36 for the solution with an initial concentration of 40 mg/L. For AC_1000°C_ MB-0.3:1, the Freundlich constant (k_f_) value was 11.13 mg/L, and the heterogeneity factor (1/n_f_) value was 0.37 for an initial concentration 40 mg/L. The Freundlich equation’s (n_f_) parameter was used to subjectively test the adsorption intensity, revealing the adsorbent’s affinity for the dye removal, and suggesting good adsorption when it had values greater than 1.

The Langmuir isotherm believes that sorption occurs within the adsorbent at different homogeneous sites. It has been successfully applied to several processes of sorption. The isotherm’s physical simplicity is based on some assumptions: Adsorption cannot occur beyond the monolayer coverage. Each site can hold only one adsorbate molecule. All sites are energetically equivalent. The surface is uniform. The linear form of the Langmuir isotherm is given by the following equation, Equation (4):

(C_e_/q_e_) = (1/Q_0_ b) + (C_e_/Q_0_) (4)

where C_e_ is the equilibrium concentration (mg/L), q_e_ is the mass adsorbed at equilibrium (mg/g), Q_0_ is the adsorbent loading (mg/g), and b is the adsorption energy (Langmuir fixed value of L/mg). The values of Q_0_ and b were determined from the slope and intercept of the linear plots’ C_e/_q_e_ versus C_e_, respectively, which resulted in a straight line of slope 1/Q_0_ corresponding to the total coverage of the monolayer (mg/g), and the intercept is 1/Q_0_b [36].

**Figure 17:**
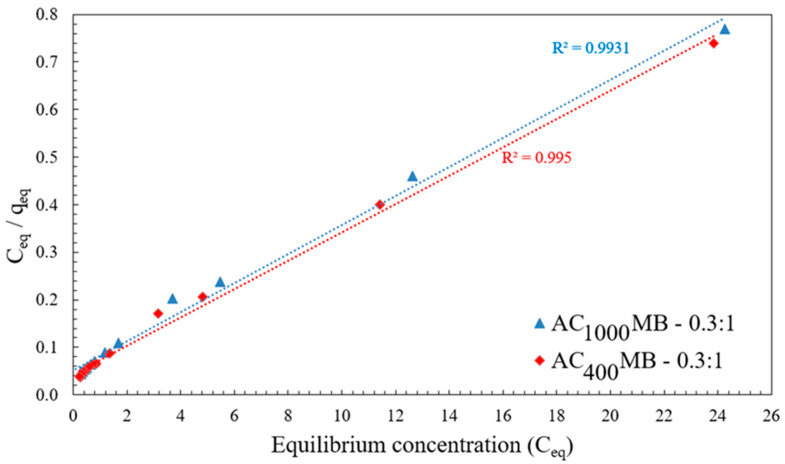
Langmuir isotherm for DB78 dye removal using AC_400°C_ MB-0.3:1 and AC_1000°C_ MB-0.3:1.

Figure 17 shows the application of equilibrium data according to the Langmuir isotherm. For AC_400°C_ MB-0.3:1, the adsorbent loading value (Q_0_) was 33.5 mg/g. The Langmuir fixed value (b) was 1.47 L/mg for an initial concentration of 40 mg/L. For AC_1000 °C_ MB-0.3:1, the adsorbent loading value (Q_0_) was 32.8 mg/g, and the Langmuir fixed value (b) was 1.68 L/mg. It is observed from the listed adsorption isothermal models in Table 2 that the adsorption of the DB78 dye using both AC_400°C_ MB-0.3:1 and AC_1000°C_ MB-0.3:1 followed the Langmuir isotherm.

### 3.4. Adsorption Kinetics

**Figure 18:**
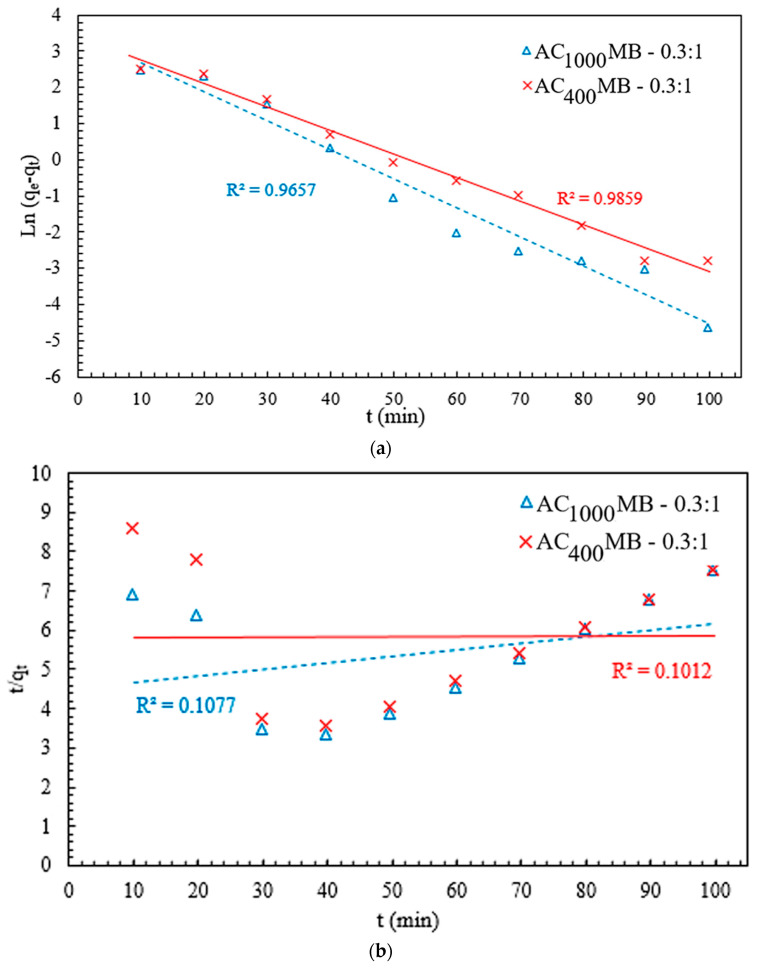
Adsorption kinetic studies: (**a**) pseudo first-order model and (**b**) pseudo second-order model.

In order to understand the mechanism of the adsorption process, kinetic studies were conducted by analyzing the samples at time intervals of 10 min until the consecutive residue dye concentrations became closer. The kinetic data for the adsorption process of the DB78 dyes AC_400°C_ MB-0.3:1 and AC_1000°C_ MB-0.3:1, with initial dye concentrations of 40 mg/L, were examined with the well-known kinetic models: the pseudo first-order model (PFO) and pseudo second-order model (PSO). The plotting of these kinetic models is shown in Figure 18.

The pseudo first-order equation.

The pseudo first-order kinetic equation was used for the adsorption analysis. The linear form of this equation is:

ln (q_e_ − q_t_) = ln q_e_ − k_1_ t (5)

where q_e_ (mg/g) and qt (mg/g) are the amounts of adsorbed adsorbate at equilibrium and at time (t), respectively, and K_1_ (min^−1^) is the rate constant of the pseudo first-order model.

The pseudo second-order equation.

The adsorption kinetics can also be described by the pseudo second-order model. The linear form of the pseudo second-order equation is expressed as:

(t/q_t_) = (1/k_2_q_e_^2^) + (1/q_e_) t (6)

where k_2_ (g/mg min) is the equilibrium rare constant of the pseudo second-order adsorption, where q_e_ (mg/g) and qt (mg/g) are the amounts of adsorbed adsorbate at equilibrium and at time (t), respectively.

Figure 18 shows the linear plots of the PFO and PSO models of AC_400°C_ MB-0.3:1 and AC_1000__°C_ MB-0.3:1. The kinetic parameters are listed in Table 3. On the basis of the low correlation coefficient for the PSO and the high value for the PFO, the adsorption abilities of AC_400__°C_ MB-0.3:1 and AC_1000__°C_ MB-0.3:1 follow the PFO rather than the PSO. These results suggest that, for AC_400__°C_ MB-0.3:1, the PFO can best predict the kinetic process. The value of q_e_ = 29.5 mg/g calculated by the PFO was more similar to the practical q_e_ = 9.76 mg/g than the PSO. For AC_1000__°C_ MB-0.3:1, the PFO can best predict the kinetic process. The value of q_e_ = 32.4 mg/g calculated by the PFO was more similar to the practical q_e_ = 9.96 mg/g than the PSO, as listed in Table 3. For AC_400__°C_ MB-0.3:1 and AC_1000__°C_ MB-0.3:1, the applicability of the PFO model indicates the interaction between the dye molecules and the microbead surface. Hence, the adsorption system is physical adsorption [37]. 

## 4. Conclusions

The removal of anionic dye (DB78) from synthetic wastewater by adsorption onto novel activated carbon/chitosan microbeads (AC_400°C_MB and AC_400°C_MB) was experimentally studied. Several microbeads with different activated carbon/chitosan ratios (0.1:1, 0.2:1, 0.3:1, 0.4:1, and 0.5:1) were synthetized in order to study the effect of the activated carbon quantity on the removal efficiency. It was observed that the removal efficiency increases with the increasing activated carbon quantity in synthetic microbeads up to a ratio of 0.3:1. The adsorption process is highly dependent on the solution pH, and the ACMB reaches its maximum equilibrium loading under acidic conditions (pH = 4:5). Based on the experimental results, it was observed that the removal efficiencies of 97.8% and 98.4% were obtained by using the adsorbents AC_400°C_ MB-0.3:1 and AC_1000°C_ MB-0.3:1, respectively, with initial concentrations of 40 mg/L. The equilibrium studies show that the initial shape of the equilibrium curve is an L shape, which means that the adsorption process resulted from the electrostatic interaction between the dye molecules and the adsorbent particles (physical forces). The adsorption studies were studied by using the Langmuir and Freundlich isothermal models, and the results were best fit to the Langmuir isotherm. Therefore, AC_400°C_ MB-0.3:1 and AC_1000°C_ MB-0.3:1 could be highly efficient sorbents for the removal of anionic contaminants. The thermochemical method using microwave radiation and an HCl solution resulted in promising dye adsorbents for textile wastewater treatment.

## Figures and Tables

**Table 1:** Adsorption characteristics for carbon samples before and after activation process.

Parameter	C_400°C_	AC_400°C_	C_1000°C_	AC_1000°C_
Average pore radius (nm)	1.67	1.63	1.78	1.57
BET surface area (m^2^/g)	32.59	99.91	33.08	138
Pore volume (cm^3^/g)	0.023	0.037	0.025	0.045

**Table 2:** Comparison of adsorption isothermal models for adsorbents (AC_400°C_ MB-0.3:1 and AC_1000°C_ MB-0.3:1).

Freundlich Isothermal			Langmuir Isothermal			Adsorbent
R^2^	1/n	K_f_ (mg/g)	R^2^	B (L/mg)	Q_0_ (mg/g)	
0.960	0.36	11.86	0.995	1.47	33.5	AC_400°C_ MB-0.3:1
0.969	0.37	11.13	0.993	1.68	32.7	AC_1000°C_ MB-0.3:1

**Table 3:** Kinetic model parameters.

Kinetic Model Parameters	Pseudo First-Order Model			Pseudo Second-Order Model		
	K_1_ (1/min)	q_e_ (mg/g)	R^2^	K_2_ (g/mg min)	q_e_ (mg/g)	R^2^
AC_400__°C_ MB-0.3:1	0.065	29.5	0.985	5.2 × 10^−5^	57.66	0.101
AC_1000__°C_ MB-0.3:1	0.08	32.45	0.965	6.11 × 10^−5^	60.24	0.107

## Data Availability

No new data were created or analyzed in this study. Data sharing is not applicable to this article.
